# Left atrial volume assessment by area-length method compared to multislice volumetric method using cardiovascular magnetic resonance imaging

**DOI:** 10.1186/1532-429X-15-S1-E97

**Published:** 2013-01-30

**Authors:** Atif Bashir, Mark Rabbat, Santanu Biswas, David Wilber, Thriveni Sanagala, Mushabbar A Syed

**Affiliations:** 1 Department of Medicine, Loyola University Medical Center, Maywood, IL, USA; 2 Heart & Vascular Institute, Loyola University Medical Center, Maywood, IL, USA

## Background

Left atrial (LA) size is a strong predictor of cardiovascular events in patients with atrial fibrillation (AF) and a variety of other cardiovascular diseases. Left atrial volume (LAV) measurement is the preferred method for assessment of LA size. The most widely used technique for LAV assessment is the area-length (AL) method using 2D echocardiography. Studies have shown that AL method by echocardiography significantly underestimates LAV when compared with CMR using multislice volumetric (MSV) method. However, MSV method is time consuming due to increased acquisition and analysis time. We sought to compare LAV assessment by CMR using the more rapid AL method with MSV method.

## Methods

We prospectively studied 273 patients with AF who underwent CMR on a 3T scanner (Siemens Trio). Image acquisition included SSFP cine of LA in short axis-stack, horizontal long-axis (4 chamber) and vertical long-axis (2 chamber) views. LAV by AL method was measured on CMR long axis views in end-systole as recommended by the American Society of Echocardiography. LAV by MSV method was performed by tracing the LA endocardial border on LA short axis stack in a phase with largest LA dimension. LA appendage and pulmonary veins were excluded by both methods. LAV and LAV index measurements by AL and MSV methods were compared using Pearson's correlation, Regression analysis and Bland-Altman plots.

## Results

CMR was successfully completed and analyzed in 252 patients; mean age 60.5 ± 10.7 years, 185 males (73.4%). Paroxysmal AF was the most common arrhythmia (57.5%). Mean CHA_2_DS_2_-VASc score was 1.7 ± 1.3. Coronary artery disease was present in 31 (12.3%), hypertension 154 (61.1%), heart failure 7 (2.8%), diabetes mellitus 34 (13.5%), TIA/CVA 18 (7.1%) and peripheral vascular disease in 7 (2.8%) patients.

Mean heart rate during CMR was 68.7 ± 15.6 beats/min, 82% were in sinus rhythm. Mean left ventricular EF was 58.6 ± 8.1% and right ventricular EF was 47 ± 7.2%.

**Figure 1:**
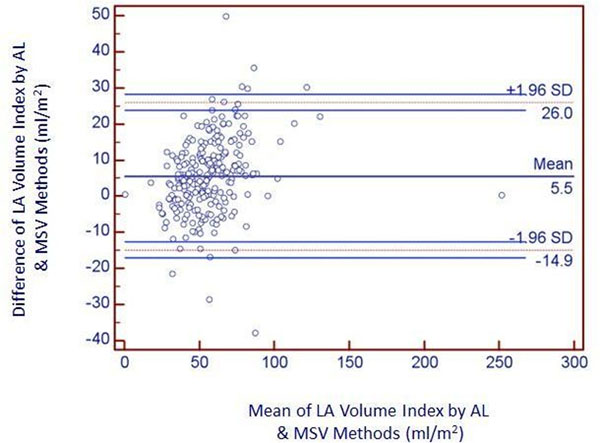
Bland-Altman plot of area-length vs. volumetric method

Table 1 shows the mean LAV and LAV index by AL and MSV methods and their correlation. LAV and LAV index by the AL method was significantly higher compared to MSV method (p <0.001 for both). AL method showed good correlation with MSV method. Bland-Altman plot showed mean measurement difference of 5.5 ± 10.5 ml/m^2^ for LAV index between two methods. AL method tend to overestimate LAV index compared to MSV method by 26 ml/m^2^ or underestimate by 14.9 ml/m^2^ (Figure 1).

**Table 1:** 

	LA volume (ml)	LA volume index (ml/m^2^)
Area-length method	123.4 ± 43.5	58.7 ± 19.8

Multislice volumetric method	111.4 ± 36.6	53.1 ± 16.2

p-value	<0.001	<0.001

Pearson correlation	0.87	0.85

R^2^	0.76	0.71

## Conclusions

LA volume measurements by CMR AL method correlate well with the MSV method but limits of agreement are wide. These differences should be taken into account when using AL method for clinical or research purposes.

## Funding

None.

